# Relapses of primary cutaneous anaplastic large-cell lymphoma in a female immunocompetent patient with persistent chlamydophila pneumoniae and human herpesvirus 8 infection

**DOI:** 10.1186/s13027-016-0079-4

**Published:** 2016-07-05

**Authors:** Elisabetta Caselli, Alessandro Borghi, Martina Maritati, Roberta Gafà, Giovanni Lanza, Dario Di Luca, Annarosa Virgili, Carlo Contini

**Affiliations:** Department of Medical Sciences, Section of Microbiology, University of Ferrara, Ferrara, Italy; Department of Medical Sciences, Section of Dermatology and Infectious Diseases, University of Ferrara, Ferrara, Italy; Department of Morphology, Surgery and Experimental Medicine, Section of Pathology and Molecular Diagnostics, University of Ferrara, Ferrara, Italy

**Keywords:** Primary cutaneous CD30+ anaplastic large-cell lymphoma, *Chlamydophila pneumoniae*, Human herpesvirus 8, Infection-related carcinogenesis

## Abstract

**Background:**

We have previously reported the case of an immunocompetent female patient with a primary cutaneous CD30+ anaplastic large-cell lymphoma (PCALCL) located on her upper right eyelid characterized by the presence of a concurrent active infection by *C. pneumoniae* and Human herpesvirus 8 (HHV8). This finding suggested for the first time a possible association of *C. pneumoniae* and/or HHV8 infection, or both together, with PCALCL pathogenesis in non-immunocompromised and HIV-negative subjects. The subsequent course of the same patient’s medical history is herein reported.

**Findings:**

During the 4 years following the surgical excision of the first PCALCL, the patient developed five further skin lesions located at different anatomical sites, all histologically proven as PCALCLs. The patient underwent several cycles of doxycycline as prophylaxis against *Chlamydia*. Skin presence of *Chlamydia* spp and HHV8 was investigated in all recurrences as well as in routine control blood samples. Amplification fragments corresponding to *Chlamydia* were found in all skin tissues analysed except one (4/5; 80 %), whereas it was not detected in any of the peripheral blood mononuclear cell samples. Conversely, HHV8 was detected in 2/5 (40 %) of the skin biopsies, including the sample negative for *Chlamydia*, but in all the blood samples analysed.

**Conclusions:**

These findings further support the hypothesis of a potential role of *C. pneumoniae* and HHV8 infection in the development and course of the described cutaneous lymphoma. A reciprocally promoting interaction between the two pathogens may be supposed to be relevant for PCALC occurrence and relapse.

## Introduction

Several infectious triggers have been associated with the development of cutaneous lymphomas. However, the actual implication of infectious agents in the pathogenesis of such disorders remains controversial [1–5]. In a previous report [6], we reported the case of an immunocompetent female patient with a primary cutaneous CD30+ anaplastic large-cell lymphoma (PCALCL) of her upper right eyelid, characterized by the presence of a concurrent active infection by *C. pneumoniae* and Human herpesvirus 8 (HHV8). This finding suggested that these microorganisms could be involved in the development of the PCALCL by reciprocally potentiating their pathogenic potential.

Here we report the subsequent course of the same patient’s medical history, with particular regard to PCALCL recurrences and concomitant search for *C. pneumoniae* and HHV8.

## Case report

The patient, a 68-year-old woman, during the 4 years following the surgical excision of the first PCALCL on her upper right eyelid (July 2011), which microbiolgical and molecular examination previously detected *C. pneumoniae* and HHV-8, presented at our Dermatology outpatient Unit for the sequential development of five further skin lesions (Table 1). For all these lesions, histopathological and immunohistochemical analyses revealed the diagnosis of cutaneous CD30+ anaplastic large cell lymphoma, like the first excised tumor [6].

**Table 1:**
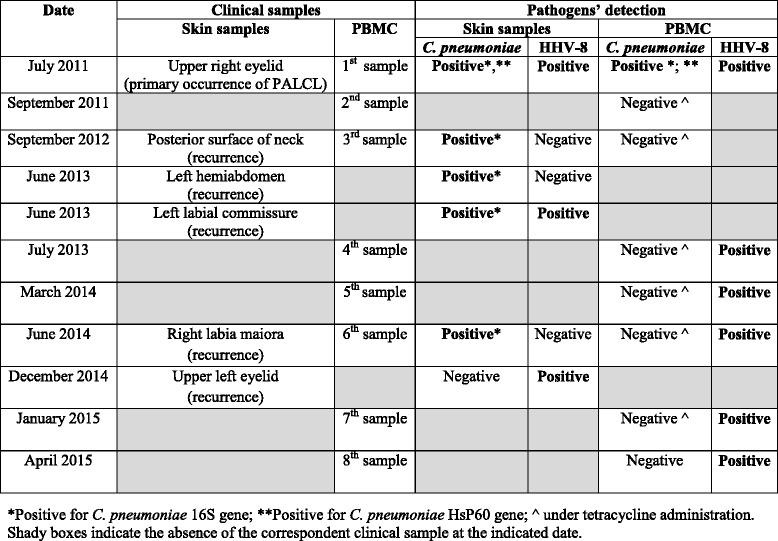
PCR results in skin specimens and PBMC from the patient with *C. pneumoniae* and HHV-8 coinfection

**Fig. 1:**
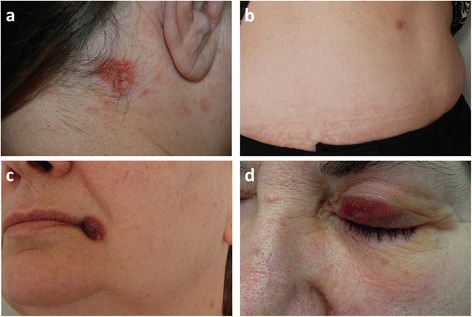
Clinical features of PCALCL recurrences located at **a**) posterior surface of the patient’s neck, **b**) left hemiabdomen, **c**) left commissure of the lips, **d**) upper left eyelid

More in detail, in September 2012 the patient presented due to the rapid onset of an erythematous, itching plaque, with a mamillated surface, 12 × 8 mm in diameter, on the right side of the posterior surface of her neck (Fig. 1a). After histological confirmation of a biopsy specimen, the lesion was treated with daily application of topical corticosteroid and oral doxycycline 200 mg/day for 3 weeks because of a possible relapse by *C. pneunoniae*. Two months later, the plaque had resolved leaving a hyperchromic macule. Then, the patient underwent three further 2-week cycles of doxycycline 200 mg/day as prophylaxis against Chlamydia, at 6–8 month intervals.

In June 2013, the woman presented with both a pinkish dome-shaped plaque on her left hemiabdomen (Fig. 1b) and a reddish-brown nodule, with well-defined borders, covered with crust, located on her left commissure of the lips (Fig. 1c). The patient referred the sudden and almost concomitant onset of both lesions, which were surgically excised.

In June 2014, an erythematous plaque, 12 × 10 mm in diameter was rapidly developed on the patient right labia maiora. The lesion was submitted to complete surgical excision.

In December 2014, the patient presented with a progressively enlarging reddish, finely scaly plaque, located on her upper left eyelid (Fig. 1d). A complete surgical excision of the lesion was performed.

It is worthy of note, that the patient referred that all the PCALCL recurrences occurred in close conjunction with stressful events or periods in her life.

Over the entire lapse of time described, periodical clinical and instrumental follow up of the patient, including positron emission tomography/computed tomography scan of the body, bone marrow biopsy, complete blood count with differential, and blood chemistry including lactate dehydrogenase, showed no evidence of extracutaneous involvement.

In June 2015, the patient addressed the hematologic unit of another hospital and was treated with gemcitabine 1000 mg/m^2^ per day, three administrations per month for a total of three consecutive cycles, due to the previous recurrences of skin lymphomas. The treatment was well tolerated by the patient. In the same period, the patient started a prophylactic treatment with isoniazid, as a latent tuberculosis infection was supposed based on both QuantiFERON test positivity and a computerized tomography scan of the chest.

Up to date, no further skin lesions appeared.

## Methods and results

Due to the microbiological findings concerning the first PCALCL lesion, skin presence of Chlamydia spp and HHV8 was investigated in all subsequent recurrences, after receiving informed consent from the patient. Both pathogens were searched in routine control blood samples as well. Approval by the institutional review board of our Hospital was not required for the present study, as materials were not collected specifically for this study, and were completely de-identified.

*C. pneumoniae* was searched in cutaneous biopsies and in peripheral blood mononuclear cells (PBMCs) isolated by density gradient from peripheral blood (Fycoll-paque plus, GE Healthcare Europe GmbH, Milan, Italy) as previously described [6, 7]. DNA was extracted and processed as previously described [7, 8], and assayed by PCR. Molecular analyses included nested-PCR using primer sets targeting 16S rRNA, outer membrane protein (ompA/MOMP), and HsP-60 genomic regions of *C. psittaci*, *C. pneumoniae*, and *C. trachomatis* [7].

In parallel, the same specimens were also used for HHV8 search, performed by two different PCRs specifically designed in the ORF50 and ORF26 genes of HHV8 genome [8]. The analysis of two different genes was undertaken to avoid the risk of false positive/negative results connected with a single gene detection.

The laboratory results are summarized in Table 1.

Amplification fragments corresponding to *Chlamydia* were found in all skin tissues analysed except one (4/5; 80 %). On the contrary, it was not detected in any of the PBMC samples. Conversely, HHV8 was detected in 2/5 (40 %) of the skin biopsies, including the sample negative for *Chlamydia*, but in all the blood samples analysed.

## Discussion

Primary cutaneous T-cell lymphoma (CTCL) are defined as lymphomas occurring at the skin level, with no evidence of extracutaneous disease at the time of diagnosis [9]. In particular, primary cutaneous CD30+ T-cell lymphoproliferative disorders are the second most common group of CTCLs and include PCALCL and lymphomatoid papulosis [10]. PCALCL occur predominantly in the adult/elderly population, where show a male predominance. They are characterized by solitary or localized nodules, tumors, or papules that frequently become ulcerated, and histologically present diffuse sheets of large tumor cells with an anaplastic, pleomorphic or immunoblastic cytomorphology, largely expressing (>75 %) CD30. Partial or complete regression may occur, but skin relapses can be observed, as in the case reported. The prognosis of PCALCL is good, with 5-year survival rates between 76 and 96 % [11].

The aetiology of CTCLs is still unclear, although a role for superantigenic activation of T cells leading to accumulation of skin-homing T cells has been suggested [12]. Notably, infectious agents have been suggested to have a role in the etiology of other lymphomas, including some non Hodgkin lymphomas, such as gastric and ocular adnexa MALT lymphomas [13–15]. In fact, some viruses can transform lymphocytes as well as a chronic immune stimulus, caused by persistent viral or bacterial infection, can be associated with the selection of lymphocytic clones potentially resulting in lymphoma development [16].

In the described patient, the initial detection of both *C. pneumoniae* and HHV8 in the first occurrence of PCALCL on her upper right eyelid, suggested a potential pathogenetic involvement of such agents [6].

Accumulating evidences suggest that *Chlamydia* spp. may play a role in oncogenesis, due to its tendency to cause persistent infections and chronic antigenic stimulation [17]. In particular, *C. pneumoniae* has been shown to be present in some CTCL and to induce the expansion of *C. pneumoniae*-specific T cells throughout the release of Sézary T cell-activating factor (SAF) [18]. Moreover, *C. pneumoniae* eradication with doxycycline has been reported to result in lymphoma regression in about a half of patients [19]. However, its implication in the pathogenesis of skin lymphomas remains unclear.

On the other hand, herpesviruses have been hypothesized as possible triggers of CTCLs [20, 21]. Among these, HHV8 seems particularly interesting, due to its oncogenic activity: it is causally related to Kaposi’s sarcoma (KS), Multicentric Castleman’s Disease (MCD), and Primary Effusion Lymphomas (PEL), and associated to other lymphoproliferative disorders occurring primarily in immunosuppresed patients [22, 23]. Interestingly, we recently observed a high prevalence of HHV8 in skin biopsies from angiomatous lesions [24], occurring prevalently in immunosuppressed individuals, suggesting that this virus might be involved in the development of skin lesions other than KS. Furthermore, several HHV8 genes can trigger cell transformation, being directly responsible for its oncogenic potential [23].

The results reported herein further support the hypothesis of a potential role of *C. pneumoniae* and/or HHV8 infection, or both together, in the development and course of the described cutaneous lymphoma. Indeed, the presence of *C. pneumoniae* was revealed in 4/5 skin PCALCL recurrences, whereas HHV8 DNA was detectable in two cutaneous recurrences only, including the one in which *Chlamydia* was not detected. On the other hand, the presence of HHV8 was a constant finding in blood PBMCs, whereas *C. pneumoniae* was no longer detectable in PBMCs after the antibiotic courses suggesting bacterial eradication from this compartment following doxycycline therapy.

The differences in pathogens’ presence in blood and tissue specimens might reflect the peculiar biological behavior of the two pathogens. In fact, similarly to other human herpesviruses, HHV8 establishes lifetime persistence in infected individuals, where 50–100 copies of virus genome per infected cell can be found in peripheral blood lymphocytes/monocytes, which represent the virus reservoir [25, 26]. Consequently, the virus load at tissue level might be lower than in blood, unless the virus is actively replicating in tissues because of reactivation.

By contrast, the reservoir of *Chlamydia* during persistence is not fully elucidated, although it has been suggested that the gastrointestinal tract might be involved [27]. Moreover, recurrent chlamydial manifestations may result from either repeated infections or persistence of the organism after unresolved but often host damaging infections, and it is not easy to differentiate between persistent infection and reinfection [28]. Accordingly, in our clinical specimens, *Chlamydia* appeared concentrated in the skin tissue, rather than in blood, where it was apparently eradicated, suggesting that its eventual presence was discontinuous and that antibiotic treatment maintained *Chlamydia* load undetectable in the blood.

Epidemiological studies have connected human herpesviruses and *Chlamydia* in several diseases, including endometritis, salpingitis, cervical cancer, chronic fatigue syndrome and multiple sclerosis [29–35]. Moreover, it has been shown that co-infection with HSV-1, HSV-2, HCMV or HHV-6 influences *C. pneumoniae* replication [36], promoting chlamydial persistence and increasing viral uptake. Interestingly, it was also reported that *C. trachomatis* induced reactivation of chromosomally integrated HHV6, and that in coinfected women low virus titers correlated with high *C. trachomatis* load and vice versa, demonstrating a potentially significant interaction of these pathogens in blood cells and in the cervix of infected patients [36]. Moreover, HHV6, CMV and HSV-1 have been reported to induce chlamydial persistence by inducing oxidative stress [36].

Our findings suggest that a similar interaction might occur also between *Chlamydia* and HHV8. The presence of HHV8 might support the persistence or the reinfection of *C. pneumoniae* in the skin, where it might induce tissue damage in spite of its apparent eradication in peripheral blood. At the same time, the presence of *Chlamydia* might facilitate reactivation of HHV8, finally resulting in the development of PCALCL recurrences. Interestingly, the appearance of recurrences following periods of psycho-emotional stress, as in the case reported, highlights the role of impairment of immune surveillance, and further support the hypothesis of a role of endogenous reactivation of the detected pathogens in the observed recurrent diseases.

A limitation of the present study is represented by the absence of transcriptional data, which would have clarified the replicative status of both pathogens in the different clinical specimens, but unfortunately the fresh specimens were not available for this analysis.

Furthermore, future mechanistic studies are needed to understand the interaction between these two pathogens inside a coinfected cell. No studies are in fact reported in the literature about the possible mutual interaction between HHV8 and *C. pneumoniae*.

Based on our observations, it would be interesting to analyze in vitro their mutual behavior in different cell types (i.e. lymphocytes/monocytes or epithelial cells) coinfected by both pathogens.

## Abbreviations

CTCLs, Cutaneous T Cell Lymphomas; HHV8, Human Herpesvirus 8; MALT, Mucosa-Associated Lymphoid Tissue; MCD, Multicentric Castleman’s Disease; PBMCs, Peripheral Blood Mononuclear Cells; PCALCL, Primary Cutaneous CD30+ Anaplastic Large-Cell Lymphoma; PEL, Primary Effusion Lymphomas.
